# A scoping review about conference objectives and evaluative practices: how do we get more out of them?

**DOI:** 10.1186/1478-4505-10-26

**Published:** 2012-08-02

**Authors:** Justin Neves, John N Lavis, M Kent Ranson

**Affiliations:** 1 McMaster Health Forum, Hamilton, Canada; 2 Bachelor of Health Sciences (Honours) Program, McMaster University, Hamilton, Canada; 3 Centre for Health Economics and Policy Analysis, Hamilton, Canada; 4 Department of Clinical Epidemiology and Biostatistics, McMaster University, Hamilton, Canada; 5 Department of Political Science, McMaster University, Hamilton, Canada; 6 Department of Global Health and Population, Harvard School of Public Health, Boston, USA; 7 Alliance for Health Policy and Systems Research, World Health Organization, Geneva, Switzerland

**Keywords:** Conferences and symposia, Conference objectives, Program evaluations, Learning and behaviour theories, Indicators of success, Evaluation methods

## Abstract

Large multi-day conferences have often been criticized as ineffective ways to improve social outcomes and to influence policy or practice. Unfortunately, many conference evaluations have also been inadequate in determining the impact of a conference on its associated social sector, with little evidence gathered or analyzed to substantiate or refute these criticisms. The aim of this scoping review is to investigate and report stakeholders’ objectives for planning or participating in large multi-day conferences and how these objectives are being evaluated. We conducted a scoping review supplemented by a small number of key informant interviews. Eight bibliographic databases were systematically searched to identify papers describing conference objectives and/or evaluations. We developed a conference evaluation framework based on theoretical models and empirical findings, which structured the descriptive synthesis of the data. We identified 3,073 potential papers for review, of which 44 were included in this study. Our evaluation framework connects five key elements in planning a conference and its evaluation (number in brackets refers to number of themes identified): conference objectives (8), purpose of evaluation (7), evaluation methods (5), indicators of success (9) and theories/models (8). Further analysis of indicators of success identified three categories of indicators with differing scopes (i.e. immediate, prospective or follow-up) as well as empirical links between the purpose of evaluations and these indicators. Conference objectives and evaluations were largely correlated with the type of conference (i.e. academic, political/governmental or business) but diverse overall. While much can be done to improve the quality and usefulness of conference evaluations, there are innovative assessments that are currently being utilized by some conferences and warrant further investigation. This review provides conference evaluators and organizers a simple resource to improve their own assessments by highlighting and categorizing potential objectives and evaluation strategies.

## Report

### Introduction

Billions of dollars are spent on large multi-day conferences every year in the hope that bringing together different stakeholders will foster collaboration and more broadly improve social outcomes [1]. Given the complexity of some of the issues discussed at these large multi-day conferences, many organizers have struggled to establish clear objectives for their conferences and ultimately, how their conferences will influence policy and practice [2,3]. Furthermore, most conferences lack comprehensive evaluation strategies and as a result, their success based on pre-determined objectives is rarely captured [4,5]. This has led numerous stakeholders to question the usefulness of such large, expensive and time-consuming conferences [3,6,7] and the quality of the associated evaluations [8]. However, before the effectiveness of these large multi-day conferences can be determined, we must first establish what the objectives of large conferences are, as defined by their stakeholders, and how they are being evaluated.

While there is a fair amount of research on the impact of smaller educational meetings, including a Cochrane systematic review [9], the study designs (randomized controlled trials (RCTs) or non-randomized controlled trials) that are typically employed by these types of evaluations may not always be feasible for large multi-day conferences. Though RCTs may provide robust conclusions regarding the effects of large conferences on social sector outcomes, political and fiscal imperatives may preclude randomization, as conference attendance often requires an invitation or abstract submission as well as adequate finances. The considerable size (sometimes thousands of attendees) and variance in the country of origin of participants also makes follow-up of outcomes logistically difficult. Finally, the budget allocated to conference evaluations is usually minimal, preventing the use of relatively expensive controlled trials (as opposed to end-of-conference surveys). Consequently, there is a significant need for robust, published research to improve the quality and effectiveness of conference evaluations [10,11].

Many studies have asserted the importance of rigorous conference evaluations, especially surrounding the inclusion of participants’ objectives and perspectives in the evaluation process [12,13], yet we found little research on how this may be achieved in practice. A few papers have studied the relationship between factors such as attendee satisfaction and intention to return and this paper will expand upon these studies by providing tangible examples of conference evaluations that have utilized such concepts [14,15]. The overall paucity of research on the subject may be due to the fact that few conferences conduct evaluations and even fewer publish their results.

The aim of this study is to explore stakeholders’ objectives for planning or participating in large multi-day conferences and how these objectives are evaluated across various social sectors. Objectives of the study are threefold: 1. to develop a practical framework connecting key elements of large multi-day conference evaluations; 2. to highlight some innovative examples of evaluations; and 3. to provide preliminary recommendations for conference organizers in building a comprehensive conference evaluation.

## Methods

A scoping review of the literature and qualitative key informant interviews were conducted to develop a practical framework for conference evaluations. An iterative approach was taken when reviewing the literature and conducting the interviews, allowing the evidence obtained to shape further research and to develop the evaluation framework simultaneously. Producing the framework concurrently with data collection allowed insight from various stakeholders to be used at all stages of its development. Methods were carried out in accordance with a study protocol completed on 25 May 2011.

### Literature review

#### Search strategy

We systematically searched eight databases from January 2000 until May 2011 in order to provide coverage across a range of social sectors. We defined "social sectors" specifically, as a broad field of work with the mandate to improve the overall wellbeing of society. Databases were selected in consultation with a WHO librarian based on previous cross-sectoral, social science literature reviews. We searched CINAHL for allied health, EconLit for economics, ERIC for education, Global Health for international public health, PAIS for public affairs/political sciences, PsycINFO for psychology, Pubmed for biomedical and life sciences, and CSA Social Services Abstracts for social sciences. A search strategy was developed based on keywords/MeSH terms of seminal papers of which we were already aware. Search strategies employed in previous reviews related to themes within the scope of this study were also consulted. Where MeSH terms could not be used, we utilized related database specific descriptors as well as basic keyword searches. The strategy was tested and reviewed with a WHO librarian and edited to include specific evaluation techniques as well as general terms (See Additional file 1 for complete search strategy). Grey literature was obtained through searching certain databases (ERIC, Global Health and PAIS), as well as other relevant sources provided by key informants. All citations were exported into Reference Manager.

#### Selection criteria

Articles included in this scoping review needed to explicitly present objectives or evaluative techniques for large multi-day conferences but this did not need to be the focus of the paper. We defined “large multi-day conferences” as a meeting lasting more than one day with at least 100 attendees. For the purposes of this review, the term stakeholders was adopted from knowledge translation literature and includes funders, researchers, knowledge brokers (i.e. conference organizers), policymakers and citizens (i.e. civil society). We excluded professional conferences of a single for-profit corporation, as goals of these conferences seem to typically focus more on profit making than knowledge exchange and sector strengthening. We also excluded online conferences and papers that solely focused on specific conference sessions. We acquired full text articles for papers that did not include an abstract or that did not specifically mention length or number of participants in the abstract, unless the citation could be excluded based on information presented in the title or abstract.

#### Data extraction and synthesis

Given the broad scope of this review and limited timeline, it was determined ex-ante that a maximum of 200 randomly selected citations from the search results in each database would be screened. Since screening was database specific, duplicates were only removed once this process was complete. Ten percent of titles/abstracts were independently screened by KR and JN and inter-rater reliability was measured using Cohen`s Kappa to determine if moving to one reviewer could be justified (kappa value, κ >.80). A single investigator (JN) conducted the data extraction, recording data on objectives or evaluation methods as well as other key characteristics (i.e. size, social sector) of large multi-day conferences presented in each of the included articles. Wherever possible, objectives or evaluations relating to a specific stakeholder group were noted. While not explicitly within the scope of this review, we tried to include any assessment of specific evaluation techniques or innovative evaluative methodologies to begin to gauge the quality of large multi-day conference evaluations. We described the data through a narrative synthesis culminating in the creation of a practical framework for conference evaluations.

### Key informant interviews

Qualitative interviews with the stakeholders (as defined in "selection criteria", above) took place throughout this project and served to both gain insight into their objectives for, and perspectives on, large multi-day conferences and to supplement the findings of the literature review. A purposive sample of 12 stakeholders from differing backgrounds was selected based on participation in one of several conferences determined through discussion with colleagues. An initial interview guide was developed and modified iteratively as new themes emerged from the scoping review or preceding interviews. Detailed notes were taken and recorded in Microsoft Word for analysis. Themes were coded as they became apparent and reviewed upon completion of all key informant interviews to ensure that themes were relevant.

## Results

### Literature review

**Figure 1:**
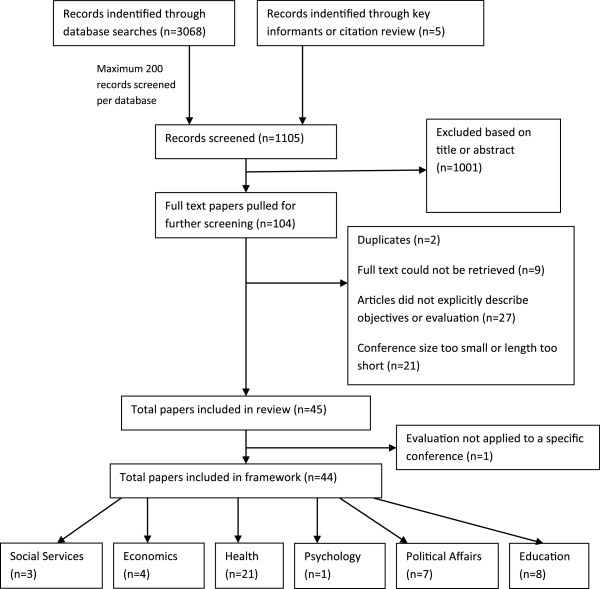
Literature search process flow chart.

Our searches identified 3,073 potential papers for review, of which 1,105 papers were screened in accordance with our predetermined cap of 200 papers per database (Global Health, PAIS, PsycINFO and Social Services each produced less than 200 references). Following title and abstract review, 113 full text papers were acquired for further screening. Two papers were excluded as they were duplicates and 57 were excluded as they were found, upon closer examination, not to meet inclusion criteria. Nine papers identified for full text review could not be retrieved and therefore, were documented for future studies and analysis but excluded from the framework (see Additional file 2). In total, 44 publications were included in this review (Figure 1).

#### Conference evaluation framework

**Figure 2:**
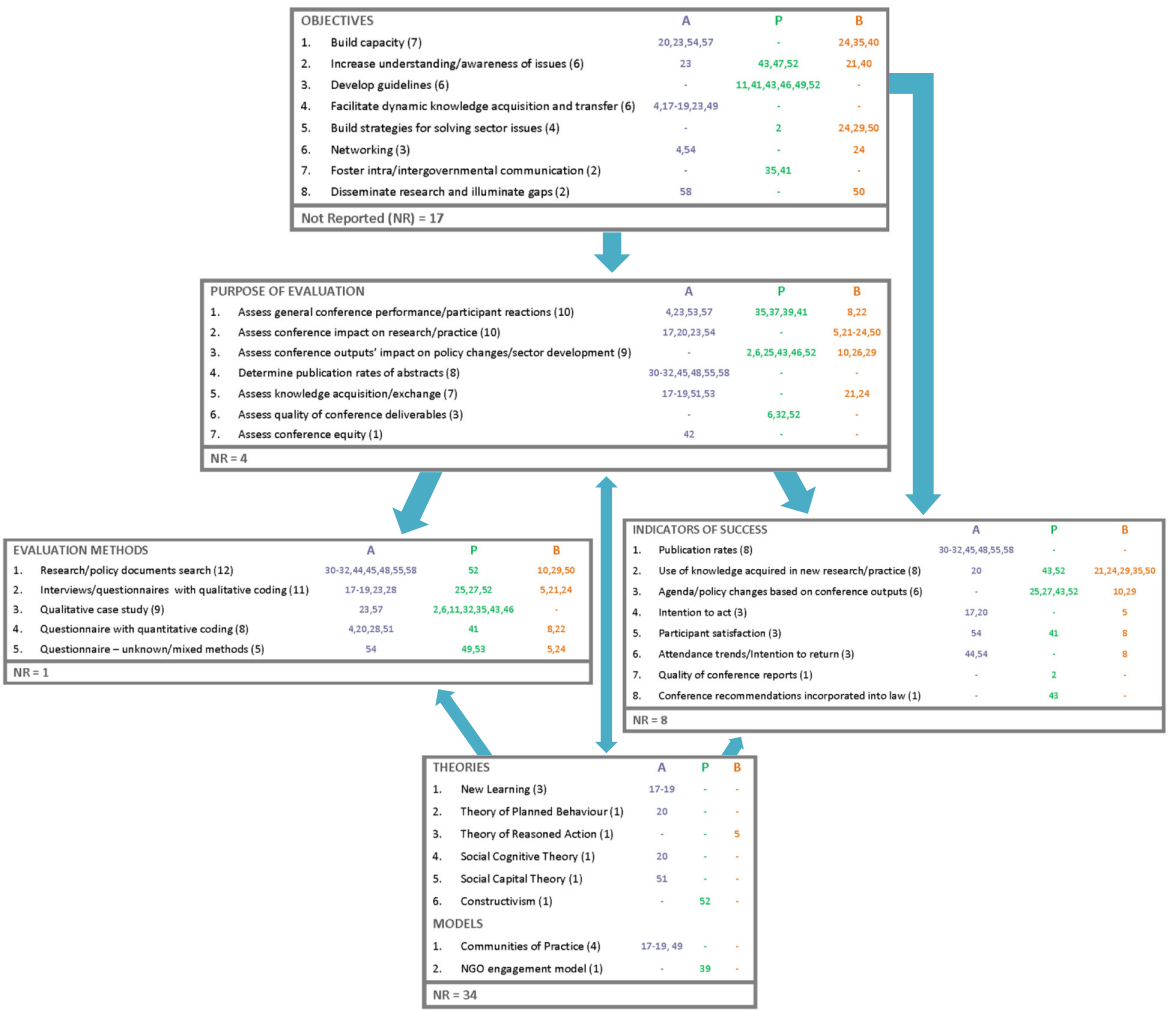
**Conference evaluation framework.** A = Academic conference, P = Political (Governmental) conference, B = Business conference. Number in brackets correspond to total articles that reported utilizing the specific objective or evaluative practice. Numbers in color correspond to article citations. Dashes are used as placeholders when no citations are coded for a category [38-60].

We iteratively developed a practical framework of key elements of a conference evaluation and incorporated our findings from the literature to provide concrete examples of what objectives and evaluative practices have been utilized (Figure 2). Our evaluation framework connects five key elements in planning a conference and its evaluation: conference objectives, purpose of evaluation, evaluation methods, indicators of success and theories/models. Given that some papers reported multiple themes for certain categories, the total number of papers in each category does not necessarily match the total number of papers reviewed.

#### Differences between conference types

Variations in evaluative strategies were found based on the type of conference being assessed, which we categorized by Rodgers’ conference definitions: academic – formal presentation of scientific research through lectures, workshops and posters, usually organized by a specific society of an academic field; political (i.e. governmental) – gathering of governmental officials and/or policymakers for discussion of issues from a political perspective; or business – private organizations facilitating conferences of various stakeholders to discuss a specific issue or emerging concern [16]. Academic and business conferences shared some overlapping characteristics (i.e. disseminating research) but business conferences tended to include a wider audience. Out of the 44 papers included in the framework, 21 were categorized as academic conferences, 13 as political conferences and 10 as business conferences. Academic conference evaluations, as a whole, tend to focus much more on learning and knowledge exchange, while political and business conferences concentrate on product creation and sector outcomes. This is particularly clear when looking at the respective objectives and indicators. In terms of specific objectives, academic conferences strongly favoured facilitating dynamic knowledge acquisition and transfer (6/21), political conferences seemed to focus on developing guidelines as the main objective (6/13) and business conferences had a fairly broad spectrum of purposes.

#### Theories/models

The authors of two papers in this review argued that including theoretical research into the development of a conference evaluation is extremely beneficial, as it helps to provide a structured framework for data collection and analysis [4,17]. In most instances, theories were used to validate the indicators being evaluated or to help determine the purpose of the assessment and ultimately, ensure that the results of the evaluation were useful. Only eight studies reported using theories in the planning of their evaluations, which are summarized in Table 1.

**Table 1:** Summary of theories/models used in conference evaluations

**Theories**	**Description**
New Learning	A theory-based tool for creating and evaluating knowledge acquisition at conferences. Weissner *et al.* define new learning as "learning that provides new insight, a diverse theoretical point of view, or a unique or uncommon conceptual framework; or points out the cumulative learning within a topic or research thread” [18].
Theory of Planned Behaviour/Theory of Reasoned Action	Two predictive behavioural theories, which posit that if: 1. a person believes a certain action (attitude) is positive; 2. their peers want them to perform this action (social pressures); and 3. they have the ability to perform this action (perceived control) then they are likely to adopt the behaviour [19]. The theory of reasoned action does not include perceived control [5].
Social Cognitive Theory	Proposes that learning can be directly related to one's observations of others, with their current cognitive processes, environment and behavioural norms acting as factors influencing overall individual development [19].
Social Capital Theory	While an accepted definition is still developing, the main concept stems from the belief that social relationships and experiences can provide positive economic and sociological outcomes for an individual and a group [20].
Constructivism	An epistemological theory that suggests humans synthesize knowledge based on the interactions of new events with previous experiences through assimilation and accommodation [21].
**Models**	**Description**
Communities of Practice	"Communities of practice are groups of people who share a concern or a passion for something they do and learn how to do it better as they interact regularly" [22]. *(quoted in Reychav and Te’eni’s study*[20]*included in the framework)*
NGO Engagement Models	Two models to describe NGOs interactions with other stakeholders and the role they play in conferences: 1. traditional lobbying of governments extending to the international stage; and 2. civil society organizations are acting independently of governments as stakeholders in global governance [23].

References directly obtained from papers included in the literature search.

Wiessner *et al.*[18] presented the only theory in this review that was created specifically for conference settings, which they entitled “New Learning”. The tool aims to provide evaluative data for the conference as well as learning opportunities through the evaluation (i.e. personal reflection) for the Academy of Human Resource Development conferences and other similar professional and academic conferences. Being a conference-specific theory, the authors incorporated conference evaluation methods based on “New Learning” principles, which were utilized in two other papers in this review [17,24]. The evaluation simply asks “What did you learn and how?” allowing participants to reflect on their own experience and share this with the organizers. The strength of this tool is in its straightforward administration and rigorous qualitative coding. The authors propose that "New Learning" changes how evaluations (mostly in an academic conference setting) are conducted and utilized, stressing the importance of "how" and "why" questions, rigorous data analysis and the dissemination of findings. Furthermore, “New Learning” is argued to be an effective guide when using broad qualitative strategies with the goal of producing results encompassing all stakeholder perspectives [17,18,24].

Jaffe, Knapp and Jeffe [19] utilized behavioural theories to create indicators specific to the purpose of their evaluation at the National Paediatric Emergency Medicine Fellows' Conference. Using the theory of planned behaviour, the team developed an end- of-conference survey to determine intentions to engage in seven targeted behaviours, instead of following up with participants in the subsequent years. Other theory-based behavioural determinants such as knowledge, social norms and confidence were evaluated to increase the robustness of results gained. Survey questions were detailed and focused, but for simplicity, required only a Likert scale (1 to 5) rating. Quantitative responses to questions such as “How likely are you to engage in new research on a topic of focus in a breakout session?” combined with information about the participants’ understanding of the topics covered, can provide insight into a conference’s impact on future research. The International AIDS Conference utilizes a similar approach based in the theory of reasoned action; a precursor to the theory of planned behaviour [5].

#### Purpose and indicators of success

**Figure 3:**
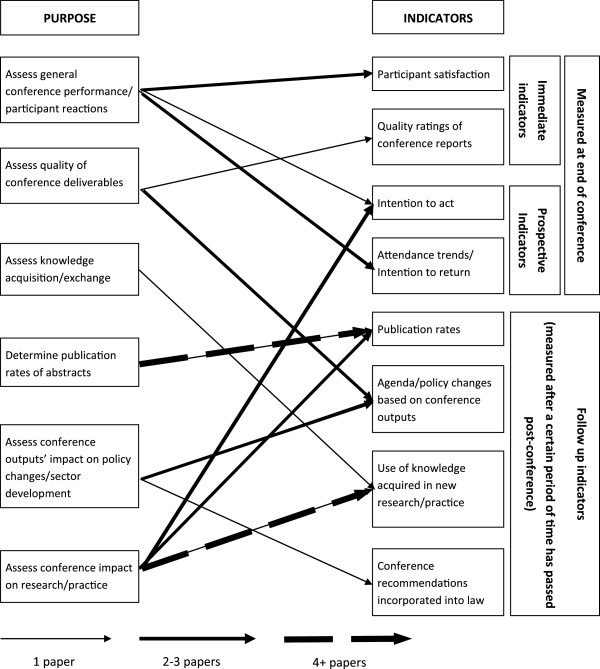
**Indicators described in relation to purpose and time of evaluation.** Arrowed lines display connections utilized by evaluations included in the review. Thicker lines correspond to a greater number of articles.

Expanding on the conference evaluation framework, Figure 3 empirically describes the links made in the literature between the purpose of an evaluation and the indicators that can be measured to help address specific goals of the assessment. The figure also breaks down indicators by immediate, end-of-conference indicators, prospective indicators and follow up indicators, which can be traced back to the purpose to help evaluators decide when is best to administer their evaluation. Publication rates were the most utilized indicator of success, likely because academic conferences were the most prominent type of conference included in the review and because of the simplicity of the evaluation. While assessing knowledge acquisition and exchange was reported as a primary goal in eight studies, there were no indicators that were directly associated with this purpose; though one paper reasoned that changes in practice post-conference may be attributed to knowledge acquired at the conference [25].

Measurement of basic indicators can also be a straightforward way to improve the usability of traditional conference surveys. General conference performance assessments, often called reaction evaluations [5] since they mainly target undeveloped reactions to events, were reported as the overall purpose of evaluations in 10 out of 44 papers (23%), while four conferences failed to identify an overall purpose for evaluation. These simple evaluations have been criticized as "opinion-level" [18] or "smile sheets" [24] and fail to address issues from participant perspectives [8]. A few studies used basic prospective indicators, such as intention to act or return, as simple additions to their surveys to add additional data on possible impact of the conference. As discussed in the theories section, many behavioural theories suggest intention as a precursor to behaviour, therefore incorporating evaluative questions based on future intentions is likely to be a good predictor of behavioural change and impact on practice. Moreover, intention to act was the only indicator to be utilized by all three conference types. There were no prospective indicators for conference impact on policy changes discovered in this review.

#### Evaluation methods

Figure 2 displays the most utilized methods by type, with searches for research and policy documents being used in 12 papers. In terms of time of evaluation, 9 out of 44 papers [5,25-32] reported utilizing follow-up surveys or interviews to either estimate the conference’s impact on stakeholders or gain feedback for future conference planning. Generally, evaluation methods seemed to be decided upon implicitly as the purpose, theories and indicators were chosen, thus it fits that a few key examples of methodologies have already been briefly presented in previous paragraphs, such as qualitative methodologies associated with “New Learning” evaluations.

James’ evaluation of the ALL WELL conference [30], was the only study to include a control group in the evaluation and one of the few to directly relate a specific impact to the conference happenings. The ALL WELL conference, promoting coordinated school health programs, conducted a follow-up evaluation in 1992 and 1999 of attendees of at least one conference between 1988–1991 and it illustrates a detailed process for evaluating sector (albeit regional) impact. Employees of 98 school districts who had participated in ALL WELL were matched with a comparison group from 98 districts without an employee in attendance. Incorporating baseline data as well as the participant- and control-group questionnaires from the 1992 and 1999 follow-up periods, the conference was able to quantitatively state that there were significantly more school wellness programs, with greater variety of activities in school districts, where employees attended ALL WELL. Due to the multiple follow-up evaluations, certain indicator trends can also be measured.

A novel publication rate analysis methodology was described in the Bank Structure Conference impact study [33]. Many of the included studies used literature (grey and/or peer reviewed) searches to assess the use of conference outputs in research or political agendas in their evaluation methods and this particular paper provides a tool to quantify conference impact in relation to the impact of associated sector journals. Using formulas modeled from journal impact factor (JIF) calculations, conference impact factor (CIF) estimates were developed. An absolute impact factor (i.e. total citations gained once conference papers are published) as well as a relative impact factor (i.e. comparison of CIFs to JIFs of prominent sector journals) was generated for each of the calculations. Literature searches took place between 2 and 26 years post-conference; however, searches were limited to articles published within two years of each conference. The study also revealed that most conference abstracts in social sciences fields take roughly five years to be published, which the authors argue should be taken into consideration when analyzing publication rates. Given the criticism of conference abstracts for poor quality and low publication rates [34-36], this tool can provide valuable evidence of these conferences' impact on future research.

A specific component of conference evaluation methods that is problematic when assessing the success or impact of large conferences is response rate, though the papers in this review offered little in the way of tangible suggestions for improvement. Overall, response rates averaged between 30 and 40%, while the number of total participants varied greatly between conferences. The authors also noted that as evaluations became more detailed in the questions asked, response rates diminished, though this may be due to lengthier evaluations.

### Key informant interviews

In total 10 interviews were conducted, with individuals identified based on participation in four different conferences. The majority of interviewees contacted had attended the First Global Symposium on Health Systems Research (Montreux 2010) but the interviews were not conference-specific and themes emerged were based on entire professional careers. Of the interviewees, 5 were organizers, 4 were researchers and 1 was a funder. Themes emerging were coded for all interviews to determine overall findings although comparisons between groups were limited to organizers and researchers. Interviews provided insight into specific experiences at conferences and how stakeholders viewed evaluations in comparison to the literature.

#### Stakeholder objectives

The major objectives from participants of large multi-day conferences are the dissemination of research, networking/professional development and increasing visibility of a specific field of work. The objectives were consistent in a large portion of the interviews though there were slight differences in primary versus secondary objectives. Organizers seemed to focus largely on dissemination of conference outputs and building awareness and understanding as primary objectives, which line up directly with findings from the literature. Networking and professional development were often discussed in the interviews but usually as an important part of any conference and not necessarily as explicit objectives from their viewpoint. Researchers tended to focus on learning objectives, such as capacity building through workshops or seminars, consistent with objectives of academic conferences, and all four researchers placed a strong emphasis on the importance of conferences as an arena for young researchers to develop and network. One funder discussed dissemination of research as a clear goal but focused on small group interactions and networking specifically by holding small group meetings as satellites to the conference.

#### Stakeholder perspectives on current evaluations

There was a dichotomy between evaluations presented in the literature and stakeholders experiences with conference evaluations. Only one of the interviewees (an organizer) was explicitly aware of an evaluation that directly influenced future conference development and none of the stakeholders had ever reported participating in any type of assessment beyond a standard reaction evaluation at the end of the conference. Furthermore, four of the interviewees had rarely or never filled out an evaluation at a conference but all were familiar with basic end-of-conference surveys.

A few key findings emerged from the majority of interviews, both in terms of the importance stakeholders placed on certain issues and how many participants independently agreed. For example, eight stakeholders referenced the importance of attendance tracking as an indicator of success. Some were specifically interested in attendance trends, including significant fluctuations, and a few mentioned analyzing participants' intention to return. There was also substantial emphasis placed on establishing diversity in a conference setting, specifically increasing the prevalence of low- and middle-income country attendees and working towards balancing the power of typically dominant groups. It is also extremely important to have “the right people attending”, as one interviewee cited the need for a mix of different levels of politicians at a conference to truly influence policy changes, for example. The interviewee provided the example that ministers need to be involved but are often too busy to participate directly in their country’s program development and implementation, so other policymakers and grassroots decision-makers should also participate in the conference, as they will be the ones actually implementing policies. Almost unanimously, stakeholders wanted to be more involved in conference planning processes to ensure that stakeholder objectives are being considered. A few such participants voiced their concern with the lack of formal conference evaluations or uncertainty as to whether current evaluations are truly being used to guide future conferences.

There was a general consensus that post-conference interviews would be the most beneficial addition to current evaluative techniques, reasoning that the impact of a conference can be established more effectively and that it allows participants to reflect in hindsight on the successes and weaknesses of the conference. One participant provided the example of always asking himself, “What did I do with all those business cards?” and a follow up evaluation would allow him to provide his feedback. A tangible example proposed by a majority of stakeholders was the implementation of follow-up telephone interviews into evaluation strategies. It was proposed that the specific time can be variable but should allow enough time for participants to have incorporated aspects of the conference into their work (approximately a year) and should take place during the planning of future conferences so that feedback can be utilized directly in this process.

What was not agreed upon was whether paper or electronic surveys were more effective as the main source of data collection. Supporters of paper submissions reported higher response rates, while proponents of electronic surveys felt that they provide the opportunity to tailor evaluations to different stakeholders. One organizer provided the specific example of using electronic surveys to provide separate surveys to specific target groups including funders and presenters. This was a strategy that many interviewees agreed was very beneficial, which was not represented in the overall conference literature.

There was little insight on specific indicators that could be used to demonstrate conference success, with all but two informants expressing concern with trying to predict the distal impact of a conference, given the myriad of possible other influences and barriers to conference impact. This being said, when asked to describe what makes a conference successful overall, achieving pre-determined conference goals and building momentum for the field were clear themes that emerged.

## Discussion

This scoping review provides a comprehensive framework of conference objectives and evaluations based on the findings of 44 included studies, supplemented by key informant interviews to gain stakeholders’ perspectives. Our conference evaluation framework connects five key elements in planning a conference assessment and identifies eight conference objectives, seven purposes of evaluation, five evaluation methods, nine indicators of success and eight theories/models described in the literature. Citations to corresponding papers in each category, separated by the type of conference evaluated, are also included in the framework (Figure 2). Figure 3 augments the framework by describing the scope of indicators of success (i.e. immediate, prospective or follow up) reported in this review and presenting empirical links between purposes of evaluations and these indicators. Key informants and the literature primarily recognized the importance of follow up components to an evaluation and prospective indicators as techniques to improve the utility of the assessment, respectively. Unfortunately, limited information was found about improving response rates for conference evaluations, however, there are multiple reviews surrounding this topic more generally [23].

Several strengths and weaknesses in this study should be considered. First, a study protocol was developed preceding the collection of data to provide a methodological framework and it was reviewed by all authors to ensure a high level of scientific rigor in the study. Second, the inclusion of multiple social databases in our search strategy also significantly adds to the strength and scope of this research, as findings of this review can be inform further investigation across a variety of social sectors. As an example of the potential for cross-sector learning, “New Learning” was developed uniquely for conference contexts and can arguably be applied to any sector. Third, through key informant interviews, we were able to gain insight into many unpublished conference evaluations and compare the theoretical strategies or evaluation “success stories” in the literature to what stakeholders want from conference assessments. One of the limitations of the study is the overrepresentation of organizers and researchers in the key informant interviews. This bias limited the comparisons between stakeholders and left out perspectives from key groups such as civil society, which is a group that some organizations have struggled to integrate into the current global governance structure. The interviewees largely came from academic backgrounds. Second, due to time constraints and limited resources for acquiring non-English texts, nine papers could not be retrieved for full text analysis, therefore potentially relevant information may have not be included in this study. These studies have been identified and cited for future research.

To our knowledge, this review is the first study to systematically analyze conference objectives and evaluation strategies across social sectors and map current practices. Previous studies, including a Cochrane review on the impact of workshops and seminars, aimed to determine the effectiveness of conferences but failed to address how current conference evaluation methodologies can be improved tangibly. Moreover, the Cochrane study largely reviewed randomized control trials focusing on continuing education conferences and healthcare outcomes whereas this study analyzed qualitative conference assessments not limited by the focus of the conference or specific outcomes.

Our framework can be used primarily as a simple tool to guide conference evaluators in selecting their objectives, methods, theoretical base etc. Secondly, it can be used to quickly identify seminal papers that may relate to any predetermined evaluation strategies that organizers or evaluators may have. It is also our hope that through this review, novel theories, study designs and methods can garner increased attention in the literature as well as in practice at conferences. For example, Ajzen, the architect of the theory of planned behaviour, has developed a tool to aid evaluators in creating questionnaires surrounding behavioural outcomes [37] but has been rarely utilized in the conference setting.

Future research is recommended concerning the evaluation of the utility of this framework. Research should assess the success of the evaluation strategies presented in this framework, in order to begin to provide concrete information on best practices. To facilitate the further analysis of the relationships between different stakeholder objectives and overall conference goals, more key informant interviews are needed with a wider variety of respondents, especially representatives of civil society. Finally, with this review being a logical first step, there must be an increase in motivation by conference planners to produce rigorous evaluations and by evaluation methodology researchers to provide tangible, accessible examples for organizers.

## Conclusions

Conference participants and evaluation researchers have criticized the lack of rigor and development in conference assessment methodologies. We hypothesized that providing a framework for organizers to efficiently acquire seminal papers related to their conference could help improve the quality of conference evaluations. This scoping review, the first of its kind, provides a valuable first step in translating research on assessments of large multi-day conferences into practice by identifying and mapping current conference objectives and evaluation strategies. Seminal examples were highlighted in the review but warrant further analysis.

## Competing interests

The authors declare that they have no competing interests.

## Authors’ contributions

JN and KR developed the initial purpose and design for the study. JN conducted the literature searches, screened possible papers for inclusion, extracted and synthesized data from included studies and wrote the manuscript (including figures and tables). KR screened 200 papers for inclusion to check inter-rater reliability and revised the manuscript drafts. JNL contributed to the development of the study’s methods and figures presented in the paper as well as revision of the overall manuscript. All authors read and approved the final manuscript.

## Supplementary Material

Additional file 1 Search strategy. Click here for file

Additional file 2 Papers identified for inclusion but could not be obtained/translated. Click here for file
