# Supplementary material for: A scoping review about conference objectives and evaluative practices: how do we get more out of them?
Source: Health Res Policy Syst. 2012 Aug 2;10:26. doi: 10.1186/1478-4505-10-26 (PMC3487916; doi:10.1186/1478-4505-10-26)
Supplement: Additional file 1 — Search strategy. [file 1478-4505-10-26-S1.doc]

**APPENDIX 1. Search strategy**

**Pubmed:**

#10 Search (((#6) OR #7) OR #8) OR #9 Limits: Publication Date from 2000 to 2012

#9 Search (#1) AND #5 Limits: Publication Date from 2000 to 2012

#8 Search (#1) AND #4 Limits: Publication Date from 2000 to 2012

#7 Search (#1) AND #3 Limits: Publication Date from 2000 to 2012

#6 Search (#1) AND #2 Limits: Publication Date from 2000 to 2012

#5 Search "Achievement"[Mesh] or "goals"[mesh] or "organizational objectives"[mesh]

#4 Search "theory of planned behaviour" or "theory of reasoned action" or "intention"[mesh

#3 Search ("Questionnaires/methods"[Mesh:NoExp] OR "Questionnaires/standards"[Mesh:NoExp] OR "Questionnaires/trends"[Mesh:NoExp] OR "Questionnaires/utilization"[Mesh:NoExp]) or "bibliometrics"[mesh] or "follow-up studies"[mesh] or "evaluation studies as Topic"[Mesh:NoExp]

#2 Search "program evaluation"[mesh] or ("Outcome and Process Assessment (Health Care)"[Mesh] OR "Outcome and Process Assessment (Health Care)/methods"[Mesh] OR "Outcome and Process Assessment (Health Care)/utilization"[Mesh])

#1 Search ((conference* [Title]) OR symposi*[Title]) OR forum[Title] or ("Congresses as Topic/organization and administration"[Mesh] OR "Congresses as Topic/statistics and numerical data"[Mesh] OR "Congresses as Topic/trends"[Mesh] OR "Congresses as Topic/utilization"[Mesh])

**Social Services:**

#4 (DE=("congresses and conventions" or "symposia") or TI=("conference*" or "symposi*" or "forum")) and(DE=("questionnaires" or "citations references") or KW="followup study")

#3 (DE=("congresses and conventions" or "symposia") or TI=("conference*" or "symposi*" or "forum")) and(DE=("action theory" or "intentionality") or KW=("planned behaviour" or "reasoned action

#2 (DE=("congresses and conventions" or "symposia") or TI=("conference*" or "symposi*" or "forum")) and(DE=("goals" or "success"))

#1 (DE=("congresses and conventions" or "symposia") or TI=("conference*" or "symposi*" or "forum")) and(DE=("effectiveness" or "evaluation" or "program evaluation"))

**CINAHL:**

#10 (S6 or S7 or S8 or S9)

#9 S1 and S5 Limiters - Published Date from: 20000101-20110531 (45)

#8 (S1 and S4) Limiters - Published Date from: 20000101-20110531 (890)

#7 (S1 and S3) Limiters - Published Date from: 20000101-20110531 (778)

#6 S1 and S2 Limiters - Published Date from: 20000101-20110531 (116)

#5 ((MH "Ajzen-Fishbein Theory of Reasoned Action") OR (MH "Intention") OR (MH "Behavioral Changes")) or AB "planned behaviour"

#4 ((MH "Questionnaires") OR (MH "Bibliometrics+")) or TI “follow up” or AB "evaluation method*"

#3 (MH "Behavioral Objectives") OR (MH "Organizational Objectives") OR (MH "Goals and Objectives") OR (MH "Success/EV")

#2 (MH "Evaluation/MT") OR (MH "Program Evaluation/MT/UT") OR (MH "Evaluation Research")

#1 ((MH "Congresses and Conferences") ) or TI conference* or TI symposi* or TI forum

**Global Health:**

#10 6 or 7 or 8 or 9

#9 1 and 5

#8 1 and 4

#7 1 and 3

#6 1 and 2

#5 questionnaires/ or follow up/ or (bibliometr$ or "evaluation methods").mp.

#4 behavioural changes/ or (intention or planned behaviour or reasoned action).mp.

#3 exp objectives/ or exp behavioural objectives/

#2 evaluation/ or program evaluation/ or health impact assessment/

#1 conferences/ or (conference$ or symposi$ or forum).m_titl.

**PAIS:**

#4 (DE= "conferences") and(KW=("behaviour change" or "theory of planned behaviour" or "theory of reasoned action" or "intention"))

#3 (DE= "conferences") and(DE=("questionnaires") or KW=("evaluation methods" or "follow up" or "bibliometr*"))

#2 (DE= "conferences") and(DE=("success") or TI=("objective*" or "goal*" or "purpose"))

#1 (DE= "conferences") and(DE=("evaluation research") or TI=("evalu*" or "effectiveness" or "impact" or "assess*"))

**PsychINFO:**

#4 (DE=("goals" or "organizational objectives")) and(TI=("Conference*" or "symposi*" or "forum" or "congress*"))

#3 (TI=("Conference*" or "symposi*" or "forum" or "congress*")) and(DE=("questionnaires" or "followup studies" or TI="evalu* methods"))

#2 (TI=("Conference*" or "symposi*" or "forum" or "congress*")) and(DE=("behavior change" or "intention" or "planned behavior"))

#1 (TI=("Conference*" or "symposi*" or "forum" or "congress*")) and(DE=("evaluation criteria" or "program evaluation" or TI=("evalu*")))

**ECONLIT:**

#4 (TI=(conference* or sympos* or forum*)) and(KW=("followup stud*" or "questionnaire*" or "bibliomet*"))

#3 (TI=(conference* or sympos* or forum*)) and(KW=("theory of reasoned action" or "theory of planned behaviour"))

#2 (TI=(conference* or sympos* or forum*)) and(TI=("objective*" or "goal*" or "success" or "purpose")) #1 (TI=(conference* or sympos* or forum*)) and(TI=(“analy*” or "evalua*" or "impact" or "effectiv*"))

**ERIC:**

#4 (DE=("conferences gatherings") or TI=(conference* or sympos*)) and(DE=("followup studies" or "questionnaires" or "content analysis" or "bibliometrics" or "evaluation methods"))

#3 (DE=("conferences gatherings") or TI=(conference* or sympos* or forum*)) and(DE=("evaluation criteria" or "program evaluation" or "summative evaluation"))

#2 (DE=("conferences gatherings") or TI=(conference* or sympos* or forum*)) and(DE=("behaviour change" or "intention") or KW=("theory of planned behaviour" or "theory of reasoned action"))

#1 (DE=("conferences gatherings") or TI=(conference* or sympos* or forum*)) and(DE=("behavioural objectives" or "organizational objectives" or "success"))
