# Supplementary material for: A scoping review about conference objectives and evaluative practices: how do we get more out of them?
Source: Health Res Policy Syst. 2012 Aug 2;10:26. doi: 10.1186/1478-4505-10-26 (PMC3487916; doi:10.1186/1478-4505-10-26)
Supplement: Additional file 2 — Papers identified for inclusion but could not be obtained/translated. [file 1478-4505-10-26-S2.doc]

**APPENDIX 2. Papers identified for inclusion but could not be obtained/translated**

1. NADEOSA 10th Anniversary Conference Formal Evaluation Report. South African Journal of Higher Education 2007:-804.

2. Cifuentes I, Bartoli SR, Miquel PM, Cabre GE, Morillas Cunill RM, Planas VR. [How many reach their goal? Outcome of abstracts presented at the XXIV AAEEH (Spanish Liver Study Association) Congress and their progress over time]. Gastroenterol.Hepatol. 2007;30(5):263-7.

3. Garcia-Muret MP, Pujol RM. Assessment of the scientific impact of presentations at Spanish National Dermatology and Venereology Congresses from 2000 through 2003. Actas Dermosifiliogr. 2009;100(1):38-45.

4. Grundmann RT, Junginger T. Symposium "Quality management in surgical oncology". Evaluation by speakers. Zentralbl.Chir 2000;125(4):398-401.

5. Laron Z. The history and impact of the International Beilinson Symposia. J.Pediatr.Endocrinol.Metab 2001;14 Suppl 1:573-4.

6. Lister ED, Pirrotta S. The annual meeting: regulatory obligation or lifeline to the future? Med.Group Manage.J 2000;47(2):42-5.

7. Pellegrini Filho A. Preliminary evaluation of Chile's First Citizen Consensus Conference. Revista Panamericana de Salud Publica 2004;15:-357.

8. Sidani S, Epstein DR. Theory-based intervention: a hallmark for generating knowledge: role of intervention theory in evaluation... 37th Annual Communicating Nursing Research Conference/18th Annual WIN Assembly, "Hallmarks of Quality: Generating and Using Knowledge," held April 22-24, 2004, Portland Marriott Downtown, Portland, Oregon. Communicating Nursing Research 2004;37:215.

9. Trudgian J. The impact of educational events needs to be evaluated. J Wound.Care 2006;15(3):102.
